# Thyroid function after diagnostic ^123^I-metaiodobenzylguanidine in children with neuroblastic tumors

**DOI:** 10.1007/s12149-022-01743-7

**Published:** 2022-05-02

**Authors:** Sarah C. Clement, Godelieve A. M. Tytgat, A. S. Paul van Trotsenburg, Leontien C. M. Kremer, Hanneke M. van Santen

**Affiliations:** 1 Department of Pediatrics, Emma Children’s Hospital, Amsterdam UMC, Amsterdam, The Netherlands; 2 Department of Pediatric Endocrinology, Wilhelmina Children’s Hospital, University Medical Center Utrecht, PO Box 85090, 3508 AB Utrecht, The Netherlands; 3 Princess Máxima Center for Pediatric Oncology, Utrecht, The Netherlands; 4 Department of Pediatric Endocrinology, Emma Children’s Hospital, Amsterdam UMC, Amsterdam, The Netherlands

**Keywords:** ^123^I-metaiodobenzylguanidine, Neuroblastic tumors, Neuroblastoma, Thyroid function, Hypothyroidism, Radiation damage

## Abstract

**Background:**

Metaiodobenzylguanidine (MIBG) labeled with radioisotopes can be used for diagnostics ^123^I^−^) and treatment (^131^I^−^) in patients with neuroblastic tumors. Thyroid dysfunction has been reported in 52% of neuroblastoma (NBL) survivors after ^131^I-MIBG, despite thyroid protection. Diagnostic ^123^I-MIBG is not considered to be hazardous for thyroid function; however, this has never been investigated. Therefore, the aim of this study was to evaluate the prevalence of thyroid dysfunction in survivors of a neuroblastic tumor who received diagnostic ^123^I-MIBG only.

**Methods:**

Thyroid function and uptake of ^123^I^−^ in the thyroid gland after ^123^I-MIBG administrations were evaluated in 48 neuroblastic tumor survivors who had not been treated with ^131^I-MIBG. All patients had received thyroid prophylaxis consisting of potassium iodide or a combination of potassium iodide, thiamazole and thyroxine during exposure to ^123^I-MIBG.

**Results:**

After a median follow-up of 6.6 years, thyroid function was normal in 46 of 48 survivors (95.8%). Two survivors [prevalence 4.2% (95% CI 1.2–14.0)] had mild thyroid dysfunction. In 29.2% of the patients and 11.1% of images ^123^I^−^ uptake was visible in the thyroid. In 1 patient with thyroid dysfunction, weak uptake of ^123^I^−^ was seen on 1 of 10 images.

**Conclusions:**

The prevalence of thyroid dysfunction does not seem to be increased in patients with neuroblastic tumors who received ^123^I-MIBG combined with thyroid protection. Randomized controlled trials are required to investigate whether administration of ^123^I-MIBG without thyroid protection is harmful to the thyroid gland.

## Introduction

Metaiodobenzylguanidine (MIBG) is a guanidine derivate and norepinephrine analogue, which is actively taken up in neuroendocrine cells via the norepinephrine transporter. Once labeled with radioisotopes it can be used for diagnostics (imaging, (^123^I^−^) or treatment (^131^I^−^) purposes in patients with neuroblastic tumors [1, 2].

Thyroid damage has been previously reported in neuroblastoma (NBL) patients who have received therapeutic ^131^I-MIBG.[3–7] This thyroid damage may present as mild thyroid dysfunction (TSH elevation) but also thyroid nodules and 3 cases with thyroid cancer have been described. This thyroidal damage most probably reflects follicular cell damage due to thyroid uptake of free circulating ^131^I^−^. Thyroid protection during exposure to ^131^I-MIBG has been proposed by administering potassium iodide (KI) (dilution and lowering uptake of the circulating ^131^I^−^). Due to the fact that KI does not protect the thyroid gland sufficiently [3], thiamazole (blocking the binding of iodide to thyroglobulin in the thyroid follicular cell resulting in a shorter exposure time to ^131^I) and thyroxine (T4) (lower uptake of ^131^I^−^ due to lowering of the serum TSH) or perchlorate (CIO4) (lowering the uptake of ^131^I^−^ by blocking the sodium-iodide transporter) can be added to KI [5–7]. Despite these preventive measures, thyroid dysfunction occurs frequently [3, 4, 7, 8], which raises the question whether the administered thyroid protection measures are sufficient or if NBL survivors are more susceptible to thyroid dysfunction regardless of given treatment. The ^131^I/^123^I-MIBG scintigraphy procedure guidelines for tumor imaging from the European Association of Nuclear Medicine (EANM) recommend the use of thyroid prophylaxis during both ^131^I and ^123^I-MIBG[9]. It may be questioned however whether thyroid protection during exposure to ^123^I-MIBG is necessary. Due to the mainly gamma irradiation, the short half-life of ^123^I^−^ and the relative low dose that is given, the expected probability of developing thyroidal radiation damage is very low after exposure to ^123^I^−^. To illustrate, ^123^I^−^ unbound to MIBG, is also used as diagnostic tool in children with suspected congenital thyroid disease to locate the thyroid gland and to objectify its function using the perchlorate discharge test [10]. In these children, the administration of ^123^I^−^ is not considered to be hazardous for thyroid function. On the other hand, although only a very small proportion of ^123^I-MIBG will circulate as free ^123^I^−^, children with NBL may have multiple courses of ^123^I-MIBG administered (up to 20 times) in the course of treatment of NBL. As it is unknown whether these repeated and higher doses might affect the function of the thyroid gland, thyroid protection is advised during exposure to ^123^I-MIBG. The aim of this retrospective cohort study was to evaluate the prevalence of thyroid dysfunction in survivors of a neuroblastic tumor who received ^123^I-MIBG for diagnostic purposes only during childhood (i.e., no treatment with ^131^I-MIBG) and to compare this with the prevalence of thyroid dysfunction in the general childhood population and with NBL patients who had been treated with ^131^I-MIBG.

## Materials and methods

### Study population

The study included all patients who were (1) diagnosed with a neuroblastic tumor (i.e., (ganglio) NBL or ganglioneuroma) in Emma Children’s Hospital, Amsterdam, The Netherlands in the period 1989–2012; (2) given ^123^I-MIBG for diagnostic purposes; (3) more than 1 year in follow-up after the last administration of ^123^I-MIBG; (4) having stable disease or being in remission after completion of therapy at the time of follow-up; and (5) did not receive ^131^I-MIBG treatment or external radiotherapy exposing the thyroid gland. Patients receiving ^131^I-MIBG for diagnostic purposes as well as patients diagnosed with preexistent thyroid dysfunction requiring levothyroxine (LT4) treatment before exposure to ^123^I-MIBG were excluded from this study. Follow-up time was defined as the period between the first administration of ^123^I-MIBG and the last measured thyroid function. Written informed consent of patients and parents was obtained prior to screening for thyroid dysfunction for the purpose of this study. The present study was approved by the local Medical Ethical Committee of the Academic Medical Center, Amsterdam, The Netherlands. Ethical approval for extra blood sampling (to test thyroid function) was denied for children < 12 years who did not need a venipuncture on clinical grounds; five children were, therefore, excluded from the study.

### Data collection

Demographic, tumor and treatment-related characteristics, as well as the frequency of ^123^I-MIBG scanning and cumulative dose of ^123^I-MIBG, and the prescribed type of thyroid prophylaxis were extracted from medical records. Disease stage was classified according to the International Neuroblastoma Staging System (INSS). Pre- and post-diagnostic TSH and free T4 (FT4) values (when available) were collected from medical records. Any documented symptoms of hypothyroidism and/or use of LT4 replacement therapy were noted. In case thyroid function was not determined more than 1 year after the last ^123^I-MIBG scintigraphy, patients were actively screened for thyroid dysfunction by measurement of plasma TSH and FT4 at the time of their regular outpatient visit. Plasma levels of FT4 and TSH were measured using standard commercial immunoassays. TSH elevation (TE) was defined as a plasma TSH concentration above the institutional age-related reference ranges independent of the serum FT4 level, or if patients used LT4 replacement therapy at the last moment of follow-up.

### Thyroid prophylaxis

To protect the thyroid gland from radiation during exposure to ^123^I-MIBG, thyroid prophylaxis was administered according to previously described protocols [5, 6]. In short, patients received 100 mg KI for 3 days during use of diagnostic ^123^I-MIBG in the time period 1989–1999. From 1999 until present, thyroid protection consisted daily of T4 100–125 μg/m^2^ in one dose and thiamazole 0.5 mg/kg body mass given in 2 doses for a period of 3 days, and KI 90 mg (100 mg per mL) was started on the morning of ^123^I-MIBG administration for a period of 2 days.

### Scan review

Radionuclide imaging in all patients was performed 24 h after injection of ^123^I-MIBG. Thyroid uptake of ^123^I^−^ was evaluated by two observers (pediatric endocrinologist and nuclear physician), who were blinded for clinical data, using a four-grade scoring system (“0” = thyroid gland not visible, “1” = faint visibility of the thyroid gland, “2” = clear visibility of the thyroid gland, “3” = not possible to assess the thyroid region appropriately or missing scan).

### Statistical analysis

Data are presented as median (range) for continues data, or n (proportion in %) for categorical variables. The prevalence of TE among survivors with (ganglio-)NBL (n = 39) was compared to the results of thyroid function measurements from two historical cohorts of NBL patients (n = 40) who had received diagnostic ^123^I-MIBG as well as ^131^I-MIBG treatment [3, 4]. Inter-group differences were evaluated using a Chi-square test, Fisher’s exact test or Mann–Whitney *U* test, depending on the type of variable. Using multivariable logistic regression analysis, we investigated the following risk factors for the occurrence of TE in the total group of NBL survivors: number of ^131^I-MIBG therapy (y/n), number of diagnostic ^123^I-MIBG administrations and chemotherapy (y/n). A *p*-value of < 0.05 was chosen to indicate statistical significance. Data analyses were performed with SPSS statistical software version 28.0 (SPSS, Chicago, IL, USA).

## Results

### Study population

In the period 1989–2012, 60 survivors with neuroblastic tumors were identified who received ^123^I-MIBG for diagnostic purposes and met all inclusion criteria. Of these, seven were lost to follow-up/followed at another institution and for 5 survivors no ethical approval was obtained for blood sampling. Forty-eight survivors were included for analysis of thyroid function. Demographic and clinical data of all included survivors are shown in Table 1.

**Table 1:** Patient demographics and clinical characteristics (*n* = 48)

Characteristic	No.	%
Sex		
Male	23	47.9
Female	25	52.1
Age at first ^123^I-MIBG (years)		
Median (range)	1.3 (0.0–16.8)	
Age at follow-up (years)		
Median (range)	11.5 (1.6–31.4)	
Follow-up time (years)		
Median (range)	6.6 (2.0–13.4)	
Histology		
Neuroblastoma	32	66.7
Ganglio neuroblastoma	7	14.6
Ganglioneuroma	9	18.8
INSS stage in (ganglio)neuroblastoma patients (*n* = 39)		
1	13	33.3
2	7	17.9
3	6	15.4
4	7	17.9
4s	6	15.4
MYCN amplification		
Yes	1	2.1
No	15	31.3
Unknown	31	66.7
Chromosome p-deletion		
Yes	–	–
No	19	39.6
Unknown	29	60.4
Number of ^123^I-MIBG		
Median (range)	1.0 (1.0–14.0)	
< 5	40	83.3
≥ 5	8	16.7
Dosage of ^123^I-MIBG (MBq/mCi)		
Median (range)	195 (83–1571)/5.3 (2.2–42.5)	
Radiotherapy^a^		
Yes	1	2.1
No	47	97.9
Chemotherapy		
Yes	14	29.2
No	34	70.8
HDCT/ASCT		
Yes	4	8.3
No	44	91.7

Data presented are median values with their range (minimum–maximum values)

*MYCN* V-myc myelocytomatosis viral-related oncogene, neuroblastoma derived [avian], *MIBG* metaiodobenzylguanidine, *MBq* megabecquerel, *mCi* millicurie, *HDCT* high-dose chemotherapy, *ASCT* autologous stem cell transplantation

^a^Radiotherapy not exposing the thyroid gland

### Visibility of the thyroid gland on the ^123^I-MIBG scans

In total 144 ^123^I-MIBG scans were evaluated for scintigraphic visibility of the thyroid gland. No thyroidal uptake of ^123^I^−^ (“0”) was seen on 109 (76%) scans, weak uptake (“1”) on 12 (8.3%) scans, and clear uptake (“2”) on 4 (2.8%) scans. On 19 (13.2%) scans, it was not possible to classify thyroidal uptake of ^123^I^−^ because of interference due to metastases or missing images. In total, on 11.1% of images in 29.2% of the survivors uptake of ^123^I^−^ was visible in the thyroid gland, despite the prescribed thyroid prophylaxis. There was no difference in the frequency of thyroidal uptake with regards to the type of prescribed thyroid prophylaxis. Of the two patients with TE, in one of the 11 images weak uptake of ^123^I^−^ was seen.

### Thyroid function

Thyroid function before administration of ^123^I-MIBG had been assessed in 14 patients, of which in 13 (92.9%) it was within the normal range. In the one patient with thyroid dysfunction before ^123^I-MIBG administration (TSH concentration 8.0 mU/L, FT4 was within the normal range), thyroid function normalized throughout follow-up. During follow-up, two survivors [prevalence 4.2% (95% CI 1.2–14.0)] developed TE. The first patient, had been diagnosed with a NBL stage 4 for which surgery and 10 courses of chemotherapy (1 × N4, 5 × N5/N6, 4 × N7) were given. The patient received 10 diagnostic ^123^I-MIBG scans throughout follow-up. Thyroid gland protection was given with a combination of KI, Methimazole and T4. On 1 of the 10 ^123^I-MIBG scans weak thyroidal uptake of ^123^I^−^ was visible. During follow-up, the boy developed TE with intermittently mild elevated TSH concentrations ranging from 6.8 to 9.6 mU/L (plasma anti-thyroid peroxidase antibodies were negative). He was treated with LT4 replacement therapy during 2 years after which he was doing well without LT4 treatment. The second patient to develop TE, had a ganglioneuroma at age 5 for which a wait and see policy was decided. The patient had received 1 diagnostic ^123^I-MIBG dose. Thyroid protection consisted of KI. No thyroidal ^123^I^−^ uptake was visible on the scintigraphic image. Thyroid function was determined 20 years after ^123^I-MIBG administration and revealed a slightly elevated TSH concentration [4.4 mU/L (reference range 0.4–3.5 mU/L)] in combination with a FT4 concentration within the normal range. The patient did not have any symptoms of hypothyroidism. Thyroid function will be monitored to exclude or confirm the diagnosis of permanent TE.

### TE in NBL patients treated with and without ^131^I-MIBG

Table 2 shows detailed information on the difference in patient demographics and clinical characteristics between a previous cohort of NBL patients treated with ^131^I-MIBG therapy (*n* = 40) and the (ganglio-)NBL survivors of the current cohort with only ^123^I-MIBG as diagnostics (*n* = 39).

**Table 2:** Patient demographics and clinical characteristics in NBL patients treated with and without ^131^I-MIBG therapy

Characteristic	^131^I-MIBG therapy *n* = 40		No ^131^I-MIBG therapy *n* = 39		*p *value
	No	%	No	%	
Sex					0.37
Male	18	45.0	22	56.4	
Female	22	55.0	17	43.6	
Age at diagnosis (years)					0.62
Median (range)	1.1 (0.0–5.2)		1.1 (0.0–11.2)		
Age at follow-up (years)					0.07
Median (range)	13.3 (4.6–22.4)		10.1 (1.6–23.3)		
Follow-up time (years)					0.02
Median (range)	11.5 (1.9–20.2)		7.5 (1.1–20.5)		
INSS stage					< 0.01
1	2	5.0	13	33.3	
2	3	7.5	7	17.9	
3	9	22.5	6	15.4	
4	21	52.5	7	17.9	
4s	5	12.5	6	15.4	
MYCN amplification					0.01
Yes	31	77.5	29	74.4	
No	7	17.5	1	2.6	
Unknown	2	5.0	9	23.1	
Chromosome p-deletion					< 0.01
Yes	10	25.0	–	–	
No	27	67.5	16	41.0	
Unknown	3	7.5	23	59.0	
Number of ^123^I-MIBG					< 0.01
Median (range)	4.0 (1.0–20.0)		2.0 (1.0–14.0)		
Number of ^131^I-MIBG					
Median (range)	2.0 (1.0–9.0)		–		
Total dosage ^131^I-MIBG (GBq/mCi)					
Median (range)	11.2 (2.0–18.6)/302.7 (54.1–502.7)		–		
Radiotherapy*					0.20
Yes	5	12.5	1	2.6	
No	35	87.5	38	97.4	
Chemotherapy					< 0.01
Yes	29	72.5	14	35.9	
No	11	27.5	25	64.1	
HDCT/ASCT					< 0.01
Yes	22	55.0	4	10.3	
No	18	45.0	35	89.7	
TSH elevation					< 0.01
Yes	17	42.5	1	2.6	
No	23	57.5	38	97.4	

Comparison of data between the survivors with (ganglio) NBL (n = 39) to the results of thyroid function measurements from two historical cohorts of NBL patients (n = 40) who had received ^123^I-MIBG and had been treated with ^131^I-MIBG

Data presented are median values with their range (minimum–maximum values)

*MYCN* V-myc myelocytomatosis viral-related oncogene, neuroblastoma derived [avian], *MIBG* metaiodobenzylguanidine, *MBq* megabecquerel, *GBq* gigabecquerel, *mCi* millicurie, *HDCT* high-dose chemotherapy, *ASCT* autologous stem cell transplantation

*Radiotherapy not exposing the thyroid gland

The prevalence of TE was significantly different between the patient groups (42.5% vs. 2.6% respectively, (*p* = < 0.01)). The patients who received ^131^I-MIBG therapy were in general more often diagnosed with high-risk NBL (i.e., higher stages NBL and/or presence of MYCN gene amplification/chromosome 1-p-deletion), required more intensive therapy (i.e., (high-dose) chemotherapy) and had received ^123^I-MIBG more frequently. Multivariable logistic regression analysis identified a significant association between ^131^I-MIBG therapy [odds ratio 33.4 (95% confidence interval: 3.8–294.5)] and the occurrence of TE (Table 3). The number of ^123^I-MIBG and chemotherapy administrations was not predictive for the development of TE.

**Table 3:** Risk factors associated with TSH elevation in NBL patients in multivariable analysis (n = 79)

Covariate	TSH elevation (*n* = 18)
	OR 95% CI
^131^I-MIBG	
No	1
Yes	33.4 (3.8–294.5)*
Total number of ^123^I-MIBG scans	0.7 (0.2–2.5)
Chemotherapy	
No	1
Yes	0.8 (0.2–3.1)

Data of the survivors with (ganglio) NBL (*n* = 39) and the NBL survivors (*n* = 40) who had been treated with ^131^I-MIBG and were previously described (results from two historical cohorts)

*OR* odds ratio, *CI* confidence interval, *MIBG* metaiodobenzylguanidine

*Significant OR’s

## Discussion

To the best of our knowledge, we are the first to evaluate the prevalence of thyroid dysfunction in neuroblastic tumor patients who had only been exposed to ^123^I-MIBG. Our results demonstrate that in this population given thyroid protection during exposure to ^123^I^−^, the prevalence of TE is very low. It may be questioned whether thyroid protection is necessary during ^123^I^−^ exposure.

Patients surviving NBL are frequently reported to have an increased risk to develop thyroid damage [13, 14]. This is most probably due to radiation exposure (i.e., radiotherapy exposing the thyroid gland and/or the administration of ^131^I-MIBG therapy) at a young age. Due to the fact that in our previous studies on ^131^I-MIBG, no correlation was found between the occurrence of TE and uptake of ^131^I^−^ in the thyroid gland, to the number of ^131^I-MIBG treatments, the received total dose, or to the age at time of diagnosis, other causes for the high percentage of TE in these survivors were considered. For this reason, it was questioned whether children with NBL have more thyroid problems irrespective of given treatment [13–15]. The data here presented do not support the idea that NBL survivors are at increased risk to develop thyroid dysfunction independent of ^131^I-MIBG treatment. We found biochemical evidence of mild TE in our study in only two patients (4.2%). When compared to the prevalence of TE or “subclinical hypothyroidism” in the general childhood population (1.7–9.5%), this seems not to be increased [11, 12]. Whether the mild thyroid dysfunction in these two survivors should be attributed to the administration of ^123^I-MIBG may be questioned. The number of patients diagnosed with TE in our study, was too small to allow for a detailed analysis regarding the independent effects of number of ^123^I-MIBG administrations, total received ^123^I^−^ dose, visible thyroidal ^123^I^−^ uptake or chemotherapy.

When our cohort was compared to a historical cohort of NBL patients treated with ^131^I-MIBG [3, 4] a significant increased prevalence of thyroid dysfunction was found for survivors after ^131^I-MIBG therapy (42.5%), indicating that ^131^I-MIBG therapy should be considered as the most likely causative factor. However, some confounders need to be considered.

First, more children in the ^131^I-MIBG therapy group received (high-dose) chemotherapy compared with the non-^131^I-MIBG therapy group. To date, the role of chemotherapy in causing TE remains unclear. Published data are conflicting; some claim chemotherapy to induce thyroid damage [16], others deny this role [17, 18]. Chemotherapy may be an additional hazard in patients who are also treated with combination radiation therapy [19]. In explorative multivariable logistic regression analysis, we did not find evidence an increased risk for treatment with chemotherapy on TE; however, the number of cases was too small to allow for strong conclusions.

Second, the follow-up time for survivors treated with ^131^I-MIBG was significantly longer when compared to the non-^131^I-MIBG group (11.5 vs. 7.5 years respectively), which may explain the relative lower incidence of TE in the ^123^I-MIBG group. However, the fact that thyroid dysfunction usually develops within the first 5 years after ^131^I-MIBG administration disproves this argument [3, 4].

Third, the fact that patients selected for ^131^I-MIBG therapy were more frequently diagnosed with high-risk NBL compared to patients who did not receive ^131^I-MIBG therapy may suggest that these patients have a different genetic profile, resulting in more advanced-stage NBL and an increased susceptibility for the development of thyroid damage, irrespective of given treatment.

The results of this study, in combination with the notion that ^123^I-MIBG has a lower irradiation risk compared to ^131^I-MIBG, strengthens the question whether administration of ^123^I-MIBG *without* thyroid protection is damaging to the thyroid gland. In accordance with the 2010 EANM Guideline, most centers administer thyroid prophylaxis for the use of diagnostic ^123^I-MIBG [9]. In 2014, Friedman et al. reported that thyroid uptake and scans for patients who received ^123^I-MIBG for cardiac imaging did not differ between patients who received thyroid prophylaxis and those who did not [20]. The authors hypothesize that the accumulation of MIBG tracer in the thyroid sympathetic nerves, rather than in the thyroid itself, resulted in the total uptake seen in the patients of whom the thyroid gland was blocked. These results were confirmed by Giubbini et al. [21] In this study, heart-failure and Parkinson patients underwent cardiac ^123^I-MIBG *with* or *without* thyroid blockade pre-treatment. Interestingly, there was no difference in thyroid parameters (thyroid/mediastinum ratio and washout) between patients who did and did not receive thyroid blockade. The authors stated that thyroid prophylaxis may not be justified in patients undergoing ^123^I-MIBG imaging as the thyroid uptake is likely a reflection of sympathetic neuronal activity. The risk of adverse effects due to pre-treatment with KI (i.e., leukopenia and the risk for iodine allergy) could be higher than the risk of exposing the thyroid gland to unnecessary radiation. Collectively, we call for long-term randomized trials that detail the efficacy and safety of thyroid prophylaxis during ^123^I-MIBG administration on thyroid function. These studies should be particularly performed in pediatric NBL patients, to answer the question whether repeated and high doses of ^123^I-MIBG during childhood are damaging to the thyroid gland.

In summary, our study confirms our hypothesis that thyroid dysfunction is not prevalent in NBL survivors after exposure to ^123^I-MIBG only. It may be questioned whether thyroid protection is necessary at all. Future studies are required to investigate whether protection of the thyroid gland is necessary during administration of diagnostic ^123^I-MIBG.
